# Effect of Agave Fructans as Carrier on the Encapsulation of Blue Corn Anthocyanins by Spray Drying

**DOI:** 10.3390/foods8070268

**Published:** 2019-07-19

**Authors:** Miguel Á. Sánchez-Madrigal, Armando Quintero-Ramos, Carlos A. Amaya-Guerra, Carmen O. Meléndez-Pizarro, Sandra L. Castillo-Hernández, Carlos J. Aguilera-González

**Affiliations:** 1 Departamento de Investigación y Posgrado, Facultad de Ciencias Químicas, Universidad Autónoma de Chihuahua, Nuevo Campus Universitario, Circuito Universitario, Chihuahua C.P. 31125, Chih., México; 2 Departamento de Investigación y Posgrado, Facultad de Ciencias Biológicas, Universidad Autónoma de Nuevo León, Ciudad Universitaria, San Nicolás de los Garza C.P. 66450, N.L., México

**Keywords:** blue corn, agave fructans, anthocyanins, encapsulation, spray-drying

## Abstract

Effects of agave fructans as carrier agents on the encapsulation of blue corn anthocyanins using spray-drying were evaluated. Blue corn extract was mixed with 6%, 8%, 10%, and 12% (*w*/*v*) of agave fructans in duplicate and dried at 150 °C. The extract showed good contents of anthocyanins, polyphenols, and antioxidant activity. The increase of agave fructans in the encapsulated powders caused a significant increase (*p* < 0.05) in the humidity, water activity (a_w_), pH, bulk density, water solubility index (WSI), and color L* values. On the contrary, the agave fructan addition decreased the hygroscopicity, water absorption index (WAI), antioxidant activity, total anthocyanin, total polyphenol, and individual anthocyanin contents. The encapsulation of blue corn extract with 6% agave fructans (*w*/*v*) resulted in good physical, thermal, morphological, and high antioxidant properties. The results suggest that the use of agave fructans as wall material represents advantages in the conservation of anthocyanins and other bioactive compounds from blue corn extract during their encapsulation. The application of blue corn anthocyanin encapsulated powders as a food ingredient is promising for food pigmentation, representing additional advantages for their contribution as a soluble fiber that can benefit the health of consumers.

## 1. Introduction

The interest in blue or pigmented corns (*Zea mays* L.) has increased in recent years due to their nutraceutical properties beneficial to the health of consumers. This is attributed to phenolic compounds, mainly anthocyanins, present in these grains [1,2]. These water-soluble pigments besides giving color, have biological activity including antioxidant, anticarcinogenic, anti-inflammatory, and neuroprotective effects, and they have been associated with the prevention of diabetes and obesity, cardiovascular diseases, and brain dysfunction, among other disorders [3,4]. The high content of anthocyanins in blue corn makes it competitive as a natural source of pigments and it can be considered as a substitute for synthetic food dyes. However, due to the instability of anthocyanins during processing and storage, their application as natural pigments in the food industry is limited [5]. Therefore, it is of general interest to apply techniques for their protection before their use in food. Microencapsulation is a promising alternative technique to improve the stability of natural pigments and protect them by entrapping with a carrier agent or wall material [6,7]. Microencapsulation by spray-drying is the most popular process used in the food industry because it is simple, fast, and can convert liquids into powders, which are easy to handle and incorporate in food formulations [8,9]. Wall materials as maltodextrin, gum arabic, and modified starch, are the most commonly used during spray-drying, which protect the sensitive compounds as anthocyanins from deterioration. Microencapsulation reduces these sensitive compounds interactions against environmental factors such as oxygen, moisture, light, temperature, and free radicals, among others [10,11,12].

Another interesting encapsulating agent is the inulin, which has been used to encapsulate bioactive compounds from cactus pear fruit [6] and blackcurrant berries [7]. Inulin-type fructans obtained from *Agave tequilana* Weber also has been used for encapsulation of astaxanthin oleoresin from *Haematococcus pluvialis* with some favorable results [9]. However, the encapsulation of anthocyanins from blue corn using agave inulin-type fructans as a carrier agent has not been reported and could represent an alternative for the conservation of anthocyanins present in these grains and be considered as a food pigment in the future. The agave fructans are composed of fructose linked through fructosyl–fructose bonds in either liner or branched form. Specifically, they consist of a complex mixture of highly branched neo-fructans with both β-(2-1) and β-(2-6) linkages between fructose moieties [13]. This configuration gives fructans a prebiotic effect and dietary fiber action because they are resistant to enzymatic hydrolysis by human digestive enzymes, and therefore pass undigested into the colon where they are fermented by the colonic microflora [14]. This prebiotic effect offers new alternative uses for agave fructans as a possible encapsulating agent for natural pigments as anthocyanins, providing food ingredients for pigmentation with functional properties to food formulations when they are added.

The objective of this research was to study the use of fructans from *Agave tequilana* Weber var. azul as a carrier agent for the encapsulation of blue corn anthocyanins by spray-drying, and to evaluate the effects on the physicochemical properties of blue corn encapsulated powders obtained at different agave fructan concentrations.

## 2. Materials and Methods

### 2.1. Materials and Reagents

Grains of blue corn (*Zea mays* L.) were obtained from the region of Babícora, Chihuahua State, México. These grains were used as a source of natural pigments (anthocyanins) for their encapsulation. Fructans inulin-type from *Agave tequilana* Weber var. azul (Agaven^®^) were provided by Nutraceúticos de Agave S. de P.R. de R.L. México (fructans ≥ 72%; DP ≥ 10). Folin–Ciocalteu’s phenol reagent, gallic acid, 2,2-diphenyl-1-picrylhydrazyl (DPPH^•^), and 6-hydroxy-2,5,7,8-tetramethylchroman-2-carboxylic acid (Trolox), cyanidin-3-glusoside, and pelargonidin-3-glucoside were purchased from Sigma-Aldrich (St. Louis, MO, USA). All other analytical grade reagents and solvents were acquired from J.T. Baker (México City, México).

### 2.2. Extraction of Blue Corn Anthocyanins

Blue corn grains were milled using a hammer mill (Pulvex model 200, México) equipped with a 2 mm sieve. Milled corn grains (100 g) were mixed with 800 mL of methanol acidified with HCl 1 N (85:15, *v*/*v*) to extract the anthocyanins. The mixture was sonicated using a probe (Branson Ultrasonics Sonifier, S-450 de 400 W Danbury, CT, USA) for 15 min and stirred in the dark during 105 min at room temperature. Then the supernatant was separated by vacuum filtration using filter paper Whatman No. 1. The extract was concentrated, removing the methanol in a rotary evaporator (Büchi Laboratoriums-Technik AG, CH-9230, Flawil, Switzerland) at 40 °C. The concentrated extract was stored at −20 °C until its use. The extraction described above was repeated until sufficient extract was obtained to carry out this study.

### 2.3. Encapsulation of Blue Corn Anthocyanin Extract

Before the spray-drying process, the frozen extract was thawed at room temperature (approximately 25 °C). Four different agave fructan concentrations, 6%, 8%, 10%, and 12% *w*/*v*, were added to 350 mL of blue corn anthocyanin extract at room temperature and mixed under magnetic agitation until dissolution. Each mix was dried in a pilot plant spray-dryer (Niro A/S Mobile Minor DK-2860 2001; GEA Company, Søborg, Denmark) with a rotary atomizer at an air pressure of 0.7 bar. The mixes were fed through a peristaltic pump (Watson Marlow 504-U; Falmouth, Cornwall, UK) and dried at 150 °C for the inlet temperature and 85 °C for the outlet temperature. The drying temperature (150 °C) was established as the most appropriate, according to some reports of anthocyanin encapsulation from different sources [7,10,15]. The spray-dried samples were immediately placed in hermetically sealed plastic bags and stored in a desiccator in the dark for further analysis. All treatments were performed in duplicate.

### 2.4. Characterization of the Encapsulated Powders

#### 2.4.1. Moisture and Water Activity (a_w_)

The moisture content was determined gravimetrically by drying in a vacuum oven at 70 °C until constant weight. Water activity (a_w_) was measured using a Novasina Labmaster-aw (Axair AG, Pfäffikon, Switzerland). Both determinations were performed in triplicate and the results were expressed as mean ± standard deviation (SD).

#### 2.4.2. Hygroscopicity

Hygroscopicity was determined according to the modified method of Cai and Corke [8]. Approximately 1 g of powder samples were placed in a container with Na_2_SO_4_ saturated solution. After equilibrium was reached (1 week), hygroscopicity was expressed as g of adsorbed moisture per 100 g of dry solids (g/100 g). This parameter was carried out in duplicate.

#### 2.4.3. Bulk Density

Bulk density (ρ_bulk_) was measured by weighing 2 g of sample and placing it in a 10 mL graduated cylinder. The cylinder was tapped by holding it on a vortex for 2 min. The bulk density was calculated as the ratio between the mass of powder contained in the cylinder and the volume occupied [16]. This parameter was performed in triplicate.

#### 2.4.4. pH

pH was determined using a pH meter (Hanna Intruments, model EDGE HI2020, Woonsocket, RI, USA) according to the method described by Sánchez-Madrigal et al. [17]. This parameter was carried out in triplicate.

#### 2.4.5. Water Absorption Index (WAI) and Water Solubility Index (WSI)

The WAI and WSI of the powders were determined in triplicate using the method described by Ahmed et al. [10] with some modifications. Powders (1 g) were weighed into centrifuge tubes, mixed with 15 mL of distilled water at 30 °C, and vigorously shaken using a vortex. The mixtures were centrifuged for 10 min at 3200× *g* (Centra CL3-R, Thermo IEC, Needhman Heights, MA, USA) and the supernatant was carefully collected into pre-weighed porcelain capsules and dried in an oven at 70 °C for 36 h. The WAI was calculated as the weight of the sediment after elimination of the supernatant between the weight of the sample. The WSI was calculated as the ratio of the dried supernatant weight between the weight of the sample.

#### 2.4.6. Glass Transition Temperature (T_g_)

The T_g_ values were determined according to Ahmed et al. [10] using a differential scanning calorimeter (TA Q-200; TA Instruments, Crawley, UK). Approximately 5–8 mg of powder was placed in a pan and hermetically sealed. The heating program increased the sample temperature from 30 °C to 120 °C followed by cooling to 30 °C at a temperature ramp of 10 °C/min under a nitrogen gas atmosphere. An empty pan was used as a reference. Thermograms were analyzed using Universal Analysis Software, version 4.7A (TA Instruments, Crawley, UK). The glass transition temperature was interpreted as the midpoint of obtained curves. Analysis was performed in duplicate.

#### 2.4.7. Scanning Electron Microscopy (SEM)

The particle morphology of the encapsulated powders was observed using a scanning electron microscope (Hitachi, model SU3500, Tokyo, Japan). Sample powders were fixed on scanning electron microscopy (SEM) stubs using 2-sided adhesive tape and coated with a gold layer under vacuum, then the samples were observed under SEM at 15 kV.

#### 2.4.8. Color

The color of the encapsulated powders was measured using a colorimeter Konica Minolta CR-400/410 (Minolta Co., Osaka, Japan), which was calibrated using a white ceramic plate. The L* (lightness), a* (greenness–redness), and b* (blueness–yellowness) parameters were determined from 10 measurements for each treatment. Hue angle (*H*° = arctan (b*/a*)), chroma (C* = (a*^2^ + b*^2^)^1/2^), and total color difference (ΔE = (($L_{0}^{*}-L^{*}$)^2^ + ($a_{0}^{*}-a^{*})$^2^ + ($b_{0}^{*}-b^{*})$^2^)^1/2^) were calculated. ΔE is the total color difference between the color of the extract and the encapsulated powders, *L*_0_*, *a*_0_*, y, *b*_0_* are the values of the extracts with the addition of fructans before the spray-drying.

#### 2.4.9. Preparation of the Extracts from Blue Corn Encapsulated Powders

Extracts used for determination of total anthocyanins, total polyphenols, individual anthocyanin content, and antioxidant activity were obtained according to the method described by García-Tejeda et al. [18] with some modifications. Accurately weighed samples (0.5 g) were mixed with 10 mL of aqueous methanol (85:15 *v*/*v* methanol/water). This dispersion was agitated for 2 min using a vortex and onicated in an ultrasound bath (Branson 1800, Danbury, CT, USA) during 20 min. The supernatant was centrifuged at 3200× *g* (Thermo IEC model CL3-R, USA) for 15 min at 4 °C and poured into a 10 mL volumetric flask and brought to volume with aqueous methanol. The obtained extracts were filtered through a syringe filter with 0.45 μm nylon membrane and stored at −20 °C for posterior analysis.

#### 2.4.10. Total Anthocyanin Content

Total anthocyanin content was determined according to that described by Li et al. [19]. The absorbance of the extracts was measured at 530 nm using a spectrophotometer (PerkinElmer model Lambda 25 UV/VIS, Perkin-Elmer, Waltham, MA, USA), as reagent blank aqueous methanol was used. The total anthocyanin content (TA) was expressed as milligrams of cyanidin-3-glucoside equivalents per 100 g of sample and was calculated on the basis of the following equation, TA = A × MW × V × 100/(ε × W), where A = absorbance, MW = molecular weight of cyanidin-3-glucoside (449.2 g/mol), V = volume of extract, ε = molar absorptivity (25,965 L/mol/cm), and W = sample weight (g). This determination was performed in triplicate for each extract.

#### 2.4.11. Total Polyphenols

Total phenolic content was determined according to the Folin-Ciocalteu colorimetric method described by Sánchez-Madrigal et al. [17]. A mixture of 30 μL of the extract, 3 mL of deionized water, and 200 μL of Folin-Ciocalteu’s phenol reagent was prepared and allowed to stand for 10 min at room temperature. 600 μL of a 20% sodium carbonate solution was added and mixed. The mixture was incubated for 20 min at 40 °C in a water bath and cooled on ice. The developed color was measured at 760 nm using a spectrophotometer (PerkinElmer model Lambda 25 UV/ VIS, USA). The absorbance of the samples was measured in triplicate using a calibration curve of gallic acid as standard. The results were expressed as mg of gallic acid equivalents per 100 g of sample (mg GAE/100 g dry basis (d.b.)).

#### 2.4.12. Antioxidant Activity

Antioxidant activity was measured using the DPPH^•^ free radical method described by Sánchez-Madrigal et al. [17]. An aliquot of 3.9 mL of 100 μM DPPH^•^ solution prepared in methanol was added to 0.1 mL of the extract. The mixture was shaken vigorously and allowed to stand at room temperature in the dark for 3 h, at which time the decrease in absorbance at 515 nm was measured using a spectrophotometer (PerkinElmer model Lambda 25 UV/VIS, USA). The results were expressed as μmol of Trolox equivalents per gram of sample (μmol TE/g d.b.). This determination was performed in triplicate for each extract.

#### 2.4.13. High-Pressure Liquid Chromatography (HPLC) Determination of Individual Anthocyanins

The extracts of the encapsulated powders prepared by duplicate were poured into amber vials for high-pressure liquid chromatography (HPLC) analysis. Individual anthocyanins were determined using an ultra-high-pressure liquid chromatography (UHPLC) system equipped with UV-visible and diode array detectors (Thermo Scientific Dionex Ultimate 3000, Sunnyvale, CA, USA). Separation was carried out on a reversed-phase Thermo Scientific Dionex C18 (5 μm, 4.6 × 150 mm) column. Aqueous 8% formic acid (solvent A) and acidified methanol with formic acid (5%, *v*/*v*) (solvent B) were used as the elution solvents. The samples were eluted according to the linear gradient for 45 min, starting with 20% to 55% solvent B in 30 min, followed by a washing and reconditioning of the column with 20% solvent B of 30–45 min. The flow rate was 1.0 mL/min, the temperature was 30 °C, and the injection volume was 40 μL. Detection was performed at a wavelength of 520 nm. The anthocyanins in the sample were identified by their retention time using the standards cyanidin-3-glucoside and pelargonidin-3-glucoside. The proportion of each anthocyanin in the extract was calculated from the percentage area of each peak in the chromatogram.

### 2.5. Statistical Analysis

A completely randomized design in duplicate was used. This was analyzed through a one-way analysis of variance (ANOVA), in which the independent variable was the percentage of carrier agent (6%, 8%, 10%, and 12%, *w*/*v*) to investigate the effect of agave fructans concentration on the final quality of the powders. Data analyses were performed using Minitab version 16 (Minitab Inc., State college, PA, USA). Differences between means across treatments were compared by Tukey’s test at a 95% significance level.

## 3. Results and Discussion

### 3.1. Characterization of Blue Corn Extract

Physical and chemical analytical results of blue corn extract are shown in Table 1. The anthocyanin content of the extract was 412.97 mg/100 g dry basis (d.b.), and in the whole blue corn was 74.36 mg/100 g d.b. The anthocyanin content in the obtained extract was similar to a purple corn extract reported by García-Tejeda et al. [18] with 426.0 mg/100 g d.b., but in whole grain was lower (94.72 mg/100 g d.b.). Also, the anthocyanin concentration for the blue corn used in this study was lower than the value reported for a blue corn from Turkey reported by Mutlu et al. [20] with 91.54 mg/100 g, but similar to a Bolivian purple corn reported by Cuevas-Montilla et al. [21] with 71.52 mg/100 g d.b., and for a purple waxy corn developed in Thailand with 75.40 mg/100 g d.b. [22]. However, it presented higher anthocyanin content than the values reported for different blue corn genotypes (34.3–14.1 mg/100 g d.b.) reported by Mora-Rochín et al. [2] and for the most of blue corn varieties developed by Urías-Peraldí et al. [1] from 37.36 to 57.44 mg/100 g. The anthocyanin content found in the extract of blue corn is acceptable because anthocyanins are only found in pericarp and in the aleurone layer, or both [23]. Therefore, the blue corn represents an important source of anthocyanins. In this way, the interest in the potential use of anthocyanins from pigmented corn as natural pigments has increased [18]. In addition, the potential that this crop represents in its yield makes it possible to obtain large amounts in relatively short times.

Regarding the polyphenol content and antioxidant activity, values of 2042.42 mg GAE/100 g d.b. and 93.57 μmol TE/g d.b. were obtained, respectively. These values are close to the values reported by Kuck and Noreña [24] for a grape skin extract with 2,626 mg GAE/100 g d.b. and 128.12 μmol TE/g d.b., and higher than that reported by Ruiz-Gutiérrez et al. [25] for a red cactus pear (*Opuntia ficus indica*) juice with 1,467 mg GAE/100 g d.b. and 63.6 μmol TE/g d.b., respectively.

### 3.2. Characterization of the Encapsulated Powders

#### 3.2.1. Moisture Content

Physical properties of blue corn encapsulated powders are shown in Table 2. The moisture content was significantly affected (*p* < 0.05) by the concentration of carrier agent (agave fructans), founding differences between 6% and 12% of fructan added with values of 9.47 and 11.07%, respectively (Table 2). As it is observed, the moisture content increased as the concentration of fructans was increased as a carrier. This is due to the fact that fructans are highly hygroscopic, and a greater quantity of these in the encapsulated powders, results in a higher affinity of fructans for water depending on the presence of greater hydrophilic groups that bind strongly to water [26], which contributes to obtaining a higher moisture content. Saénz et al. [6] reported higher moisture content in microcapsules of red cactus pear bioactive compounds added with inulin. Also, de Barros Fernandes et al. [26,27] reported an increase in moisture content with the addition of inulin in encapsulated of rosemary essential oil with different carriers.

The moisture content found in this study for all encapsulated powders is slightly high compared to that reported by other researchers [6,25]. According to Pitalua et al. [28] the moisture content depends on the carrier agent and the drying conditions. These two factors could be responsible for the moisture content found in this study, since only agave fructans in different concentrations were used as carrier agent, which are highly hygroscopic and could favor the absorption of moisture in the encapsulated powders. On the other hand, the spray-drying was carried out at 150 °C, which prevented a greater elimination of water in the powders of the different treatments, also contributing to high moisture contents.

#### 3.2.2. Water Activity (a_w_)

Water activity is considered one of the factors that most influence food safety and stability, a_w_, with temperature, controls the physical and chemical properties of powders [28]. Water activity showed significant differences (*p* < 0.05) between the encapsulated powders, but only between the powders with 6% and 12% of fructan addition (Table 2). These treatments presented values of 0.123 and 0.156, respectively. The found trend for a_w_ was similar to the moisture content, demonstrating that greater concentration of fructans generate higher hygroscopic properties.

However, all the powders obtained are within the range reported by other authors during the encapsulation of different pigments and bioactive compounds with different carriers [9,18]. With these a_w_ values (Table 2), the pigments stability is safe due to the minimization of biochemical deterioration and microbial growth [29]. This is because in dry atmospheres the water is strongly attracted to the polar sites on the surface of the microcapsules, and therefore, it is not available for any type of reaction [28].

#### 3.2.3. Hygroscopicity

For the hygroscopicity of the encapsulated powders, a significant effect was found (*p* < 0.05) between fructan percentages without differences between 10% and 12% (Table 2). These hygroscopicity values ranged from 38.17% to 49.68%, with the highest value in the powders with the lowest percentage of fructans added (6%). At lower moisture content the hygroscopicity was higher (Table 2). This same behavior was reported by Tonon et al. [11] and Otálora et al. [30]. According to these authors, the powders with lower moisture content have a greater capacity to absorb water from the environment due to a higher water concentration gradient between the product (encapsulated) and the surrounding air. De Barros Fernandes et al. [26] found that the presence of inulin as a carrier agent decreased the hygroscopicity during storage of powders at high relative humidity. The hygroscopicity values found are similar to those reported by Cai and Corke [8] and lower than those reported by Ersus and Yurdagel [31].

#### 3.2.4. Bulk Density

Bulk density of the powders ranged from 0.6254 to 0.6477 g/cm^3^ (Table 2), showing a slight tendency of higher values at higher concentration of fructans, but only the powders with fructan addition of 6% and 12% presented significant differences (*p* < 0.05). According to Tonon et al. [32], the bulk density of the encapsulated is related to the molecular weight of the carrier materials used, therefore the heavier materials, the more easily it accommodates in the spaces between the particles occupying less space and resulting in higher bulk density values. The aforementioned could have occurred due to the effect of a greater concentration of fructans added. These results (Table 2) also can be associated with the higher moisture content obtained at these conditions, since Chegini and Ghobadian [33] reported that powders obtained by spray-drying with higher moisture content tended to have a higher weight by volume due to the presence of water, which is considerably denser than the dry solid. In addition, Rajam and Anandharamkrishnan [16] reported higher bulk density values in encapsulated powders containing only fructooligosaccharides (FOS) than with the other carriers used, this is attributed to the aggregation of the particles and to less space between them. Bulk density values found in this study are within the ranges reported by other authors in encapsulated powders of pigments with different carriers [12,25,32], satisfying with an important characteristic for their handling, storage, and processing.

#### 3.2.5. pH

pH values of the encapsulated powders are shown in Table 2, which presented significant differences (*p* < 0.05) between the percentages of fructan addition. As it is observed, the pH’s of the encapsulated powders are low because the starting extract presented a pH of 1.79. However, the pH increased slightly with the increase of fructans, presenting the encapsulated with 12% the highest pH value (2.23). This is due to the increase of carrier concentration with pH of 5.12. As already mentioned, although the powders presented low pH values, these are suitable for anthocyanin stability [3].

#### 3.2.6. Water Absorption Index (WAI) and Water Solubility Index (WSI)

Both, WAI and WSI were significantly affected (*p* < 0.05) by the addition of fructans in the blue corn encapsulated powders. For the WAI, all concentrations were statistically different and for the WSI only 12% of fructan addition did not present differences with the rest. Table 2 shows that the WAI decreased and conversely, the WSI increased with the increase of fructan concentration. Also, it is observed that non-encapsulated powders (carrier) presented lower WAI and higher WSI than the encapsulated powders, these differences can be attributed to the fructan hydrolysis due to the strong acidity of the extract [34], increasing the absorption and decreasing its solubility. Ahmed et al. [10,35] reported decreases in the WAI and increases in the WSI by increasing the concentration of the carrier (maltodextrin). The obtained WAI results are related to hygroscopicity, which presented the same trend. The increase in the WSI may be due to the fact that at high fructan concentrations the particles are closer, and this interaction can form agglomerates, obtaining smaller WAI and subsequently higher WSI values [10,26]. According to Syamaladevi et al. [36], the solubility is considered as an important instantaneous property (wettability, dispersibility, solubility) for encapsulated powders, since they must be rehydrated when they are used as a food ingredient. The WSI found in all encapsulated powders are acceptable, and result in easy-to solubilize powders, this is because their components such as pigments, sugars, and carrier have a high-water solubility.

#### 3.2.7. Glass Transition Temperature (T_g_)

The T_g_ values of encapsulated powders were not affected (*p* > 0.05) by the concentration of fructans as a carrier, ranging from 50.01 to 53.55 °C (Table 2). These T_g_ values found for this study are favorable, since storage temperatures higher than T_g_ provide greater molecular mobility by accelerating reaction rates. Conversely, temperature conditions below the T_g_ provide greater stability of the products against deterioration during storage. Therefore, an encapsulated powder product should be stored at a temperature below its T_g_ value [25]. The T_g_ values found demonstrate that agave fructans have some potential for application as wall material during encapsulation of bioactive compounds for the food industry.

#### 3.2.8. Color

**Figure 1:**
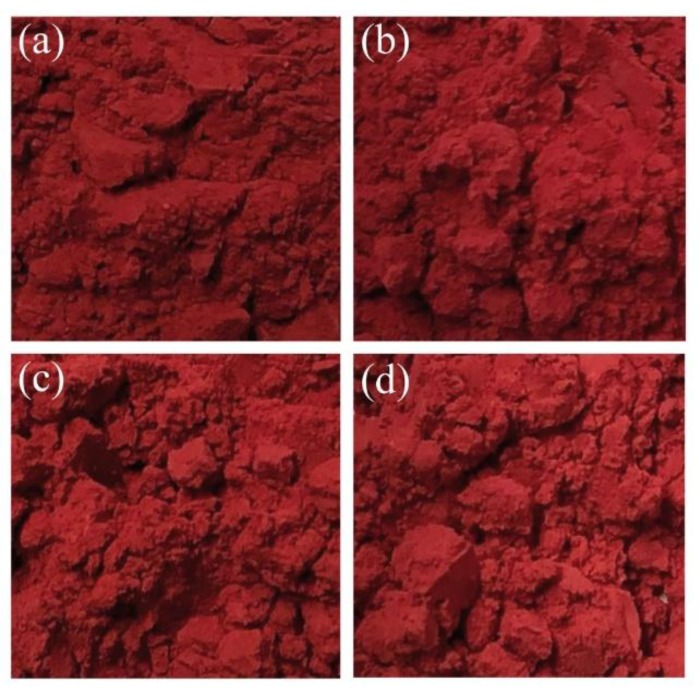
Encapsulated blue corn powders obtained by spray-drying at different addition of agave fructans. (**a**) 6%, (**b**) 8%, (**c**) 10%, (**d**) 12%.

The color is an important characteristic during the encapsulation of pigments, since one of the functions of the encapsulated is to impart color or pigment food. Figure 1 shows the color obtained in the encapsulated powders with different concentrations of agave fructans. The color parameters of powders are shown in Table 3. The parameter L*, which represents lightness, was significantly affected (*p* < 0.05) by the percentage of fructan addition. Increases in the concentration of fructans resulted in higher L* values, which could be due to a whitening tendency from the agave fructans. This effect is observed in Figure 1, where a slight increase in luminosity is noticeable as the amount of agave fructans was increased. Similar results were reported by Kha et al. [29] for gac fruit juice powder and by Ruiz-Gutiérrez et al. [25] for red cactus pear powder, using maltodextrin and β-glucans as carrier agents, respectively.

The parameter a* was not significantly (*p* > 0.05) affected by fructan concentration. The positive values from 36.25 to 36.33 indicate a trend to redness, which is also appreciated in Figure 1. Regarding to parameter b*, it was not significantly (*p* > 0.05) affected by the concentration of fructans added. For chroma* and hue angle, which indicate the color intensity (or saturation) and the color purity respectively, no statistical differences (*p* > 0.05) were observed between the different encapsulated powders. The chroma* showed values of 38.73 to 38.9, while the hue angle showed values near 0°, indicating a pure red color of the powders. The found results of parameter a* and hue angle are representative of anthocyanins color at low pH. Additionally, the total difference of color (ΔE) was obtained, showing a significant effect (*p* < 0.05) between fructan concentrations (Table 3), mainly affected by lightness (L*), as already mentioned.

#### 3.2.9. Scanning Electron Microscopy (SEM)

**Figure 2:**
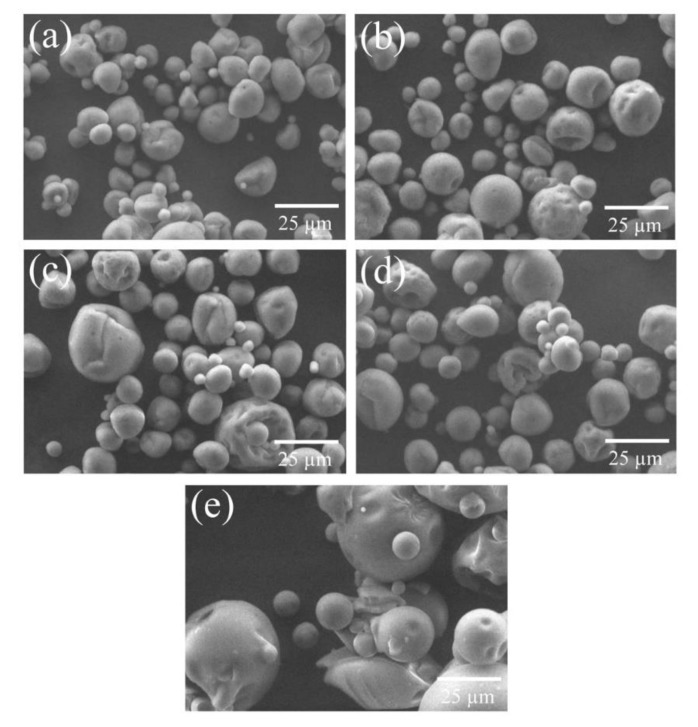
Micrographs of encapsulated blue corn powders obtained by spray drying at different agave fructan addition. (**a**) 6%, (**b**) 8%, (**c**) 10%, (**d**) 12%, (**e**) agave fructans. 1000×.

SEM analysis of encapsulated powders is shown in Figure 2. The obtained micrographs showed a matrix of capsules with spherical shape and smooth surface. Some collapsed or compressed capsules with some agglomeration between particles were observed. Also, the particle size of capsules (5–30 μm) were similar in all treatments, indicating that different concentrations of fructans did not influence in the microstructure of powders. The only difference in particle size was observed in agave fructans (non-encapsulated) with larger and less-compressed spheres (Figure 2e), only with small marks due to the arrangement between fructan particles.

The morphology of encapsulated powders with agave fructan as a carrier was typical of a spray-drying encapsulation process. These non-spherical or irregularly shaped capsules with the appearance of cavities in the surface, are attributed to wall collapse due to rapid water evaporation and consequent contraction of the particles during the drying process [6,12,37,38]. The deformation of the spherical shapes found in all the treatments was probably due to the evaporation temperature of 150 °C used, since for low spray-drying temperatures, longer exposure times are required, causing deformation, shrinkage, and collapse of the capsule structure [37]. Similar morphologies were observed in microcapsules of bioactive compounds of red cactus pear with maltodextrin and inulin [6], as well as in encapsulated of astaxanthin oleoresin from *Haematococcus pluvialis* with gum arabic and inulin of *Agave tequilana* Weber [9], and in encapsulated of rosemary essential oil with gum arabic, modified starch, and inulin [27].

#### 3.2.10. Total Anthocyanin Content

**Figure 3:**
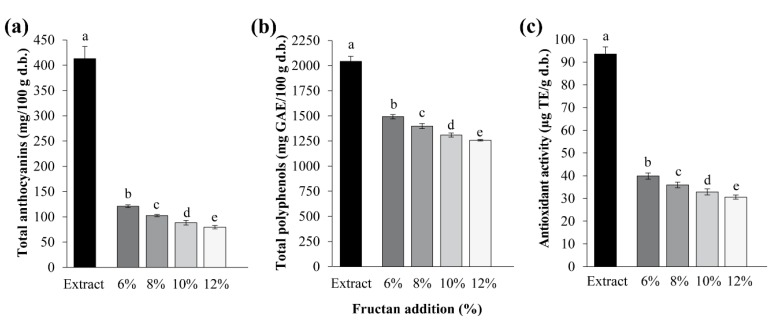
(**a**) Total anthocyanin content, (**b**) total polyphenols, (**c**) antioxidant activity in encapsulated blue corn powders obtained by spray-drying at different agave fructan addition.

Chemical analysis of encapsulated powders of blue corn extract showed that the total anthocyanin content ranged from 120.87 to 79.61 mg/100 g d.b. and it was significantly affected (*p* < 0.05) by the concentration of agave fructans, decreasing with the increase of this carrier agent (Figure 3a). This behavior is related to the increase in the total solids content caused by the addition of agave fructans. This result agrees with that reported by Ahmed et al. [10,35] in purple sweet potato flours and encapsulated respectively, using α-amylase, maltodextrin, and ascorbic acid as carriers. Another factor related to the decrease in anthocyanin content by the addition of fructans could be attributed to the spray-drying conditions used, producing encapsulated powders with high moisture content and a_w_ values (Table 2). Therefore, the encapsulated anthocyanins could degrade, because their main structure (cation flavylium) could hydrate and generate to the colorless chalcone structure [39]. The loss of anthocyanins by effect of high humidity has been reported by several researchers [18,38]. Bakowska-Barczak and Kolodziejczyk [7] reported that with the use of inulin as a carrier, the total anthocyanin content decreased significantly compared with those obtained only with maltodextrin, attributing it to the greater inulin hygroscopicity being more susceptible to the degradation of these colorful compounds.

When comparing the encapsulated powders with the blue corn extract, a significant loss (*p* < 0.05) in total anthocyanin content due to its susceptibility to temperature caused by spray-drying was observed (Figure 3a). The degradation of anthocyanins at high temperatures may be due to the hydrolysis of the 3-glucoside structure, which has a protective effect on the unstable anthocyanin, or by the hydrolysis of the pyrilium ring resulting in chalcones, which are responsible for brown colorations [39,40]. A greater anthocyanin loss in encapsulated powders at higher temperatures in the spray-dryer has already been reported [7,31]. The retention percentages of anthocyanins varied from 29.27% to 19.27% for the encapsulated with lower and higher fructan concentration, respectively.

High anthocyanin contents in encapsulated powders from different sources have been reported [7,24,31]. Similar contents in powders from fruit (*Bactris guineensis*) with maltodextrins (118 to 131 mg/100 g) were reported by Osorio et al. [38]. Also, lower contents have been reported by García-Tejeda et al. [18] in encapsulated powders from purple corn extract using corn starch as a carrier (46–85 mg/100 g), by Robert et al. [41] in encapsulated from pomegranate extract with 47 to 84 mg/100 g, and by Ahmed et al. [10] in purple sweet potato encapsulated (52 to 57 mg/100 g). These differences between studies are mainly since the sources used are rich in anthocyanins. In addition, their composition regarding the type of anthocyanins and their proportion, the type of carrier, as well as the drying conditions used to encapsulate, as already mentioned, are factors that affect the anthocyanin final content.

#### 3.2.11. Total Polyphenols

The analysis of the encapsulated powders showed that the polyphenols content ranged from 1491.70 to 1257.87 mg GAE/100 g d.b. and it was significantly affected (*p* < 0.05) by the amount of agave fructans added to the blue corn extract. As the percentage of fructans increased, the total polyphenol content decreased (Figure 3), which is also related to the increase in the solids content originated by fructans. This result agrees with Ruiz-Gutiérrez et al. [25] who found lower content of polyphenols in red cactus pear juice capsules by adding more soluble fiber content (β-glucans).

Comparing the encapsulated powders with the extract, a significant decrease (*p* < 0.05) was observed (Figure 3b). These results indicate that polyphenols are lost during the spray-drying process. This loss is related to the susceptibility of some phenolic acids to degradation and polymerization during exposure to high temperatures and oxygen [12]. Regarding the effect exerted by the fructan addition, the retention percentages ranged from 73.03% to 61.58% for the encapsulated with lower and higher fructan concentration, respectively. The phenolic content of powders is similar to that found by Bakowska-Barczak and Kolodziejczyk [7] in blackcurrant encapsulated at 150 °C with dextrose (1163.5 to 1251.4 mg GAE/100 g) and inulin (967.2 mg GAE/100 g) as carriers. Saikia et al. [12] quantified lower values of total polyphenols (535 to 825 mg GAE/100 g) in capsules of *Averrhoa carambola* pomace with maltodextrin. Also, Robert et al. [41] reported minor polyphenol content in pomegranate powders (284 to 151 mg GAE/100 g). As can be observed, the encapsulated powders of blue corn extract presented a good polyphenol content compared with other studies. Obtaining the highest phenolic content (1491.70 mg GAE/100 g) in the powders with 6% of fructans added.

#### 3.2.12. Antioxidant Activity

The addition of agave fructans significantly affected (*p* < 0.05) the antioxidant activity of the encapsulated powders of blue corn extract. A higher fructan addition caused less antioxidant activity in the powders, varying from 39.84 to 30.57 μmol TE/g (Figure 3c). Ahmed et al. [10] reported similar results, finding lower antioxidant activity in encapsulated powders with higher concentration of ascorbic acid and maltodextrin as carriers. Figure 3c shows how the spray-drying method significantly affected (*p* < 0.05) the antioxidant activity of the encapsulated powders, showing a marked decrease with respect to the initial extract, resulting with retention percentages of 42.57% to 32.67% for the encapsulated with lower and higher fructan concentrations, respectively. Ruiz-Gutiérrez et al. [25] reported during the encapsulation of red cactus pear compounds that the antioxidant activity was not significantly affected by the spray-drying conditions. However, Kuck and Noreña [24], during the encapsulation by spray-drying of compounds from skin of grape variety Bordo, reported that antioxidant activity decreased between 42.7% and 53.07%. The obtained results in antioxidant activity may be due to the fact that blue corn components such as anthocyanins are more susceptible to high drying temperatures [7,31]. This was confirmed with the correlation analysis between the values of total anthocyanins, total polyphenols, and antioxidant activity of the encapsulated powders (Table 4), where these three determinations were significantly correlated with values of *R* = 0.933−0.951. Sánchez-Madrigal et al. [17] reported a high correlation of antioxidant activity with anthocyanin and polyphenol contents in blue corn tortilla chips. Ahmed et al. [10] reported in encapsulated powders of purple sweet potato the correlation between anthocyanin content and antioxidant activity. According to Suda et al. [42], at least one caffeoyl group acylated to anthocyanins contributes to high radical-scavenging activity.

#### 3.2.13. Individual Anthocyanins

**Figure 4:**
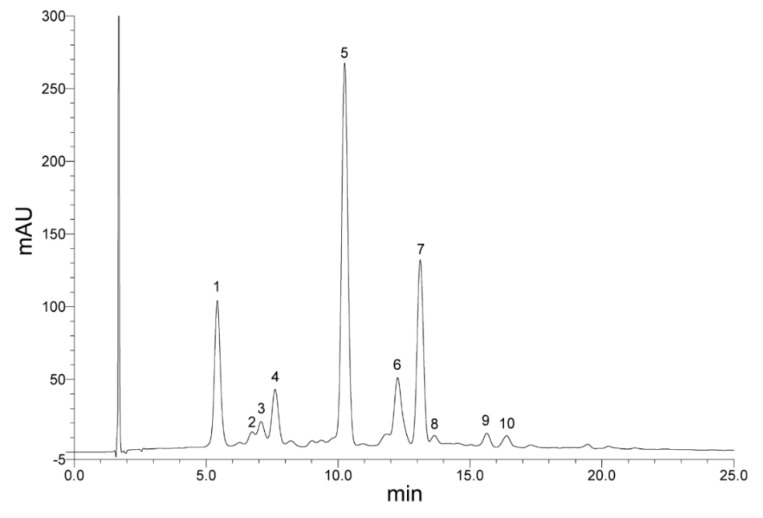
High-pressure liquid chromatography (HPLC) chromatogram of blue corn extract anthocyanins. Peak 1: cyanidin-3-glucosise, peak 3: pelargonidin-3-glucoside, peaks 2, 4, 5, 6, 7, 8, 9, 10: not identified.

Figure 4 corresponds to the chromatogram of anthocyanins present in the blue corn extract. Cyanidin-3-glucoside (peak 1) and pelargonidin-3-glucoside (peak 3) were identified by their retention times with concentrations of 215.50 mg/100 g d.b. and 31.70 mg/100 g d.b., respectively. Additionally, several unidentified anthocyanins were detected, as peaks, 2, 4, 5, 6, 7, 8, 9, and 10. The proportion of the identified anthocyanins cy-3-glu and pg-3-glu was 15.74% and 4.97%, respectively. However, 2 peaks were found with higher proportions than cy-3-glu: peak 5 with 39.25% and peak 7 with 17.88%, which may be acyl-type anthocyanins, as well as 2 peaks higher than pg-3-glu: peak 4 with 7.66% and peak 6 with 9.35%. Acylated anthocyanins in similar chromatograms of pigmented corns have been reported [18,20,22].

According to the chromatograms of anthocyanins for a purple corn reported by Cuevas-Montilla et al. [21] and Lao and Giusti [15], the unidentified anthocyanins found in this study as peaks 4, 5, 6, and 7 could be peonidin-3-glucoside, cyanidin-3-(6′′-malonylglucoside), pelargonidin-3-(6′′-malonylglucoside), and peonidin-3-(6′′-malonylglucoside), respectively. In addition, according to the chromatogram reported by García-Tejeda et al. [18], the peak 2 could be cyanidin-3-rutinoside.

**Figure 5:**
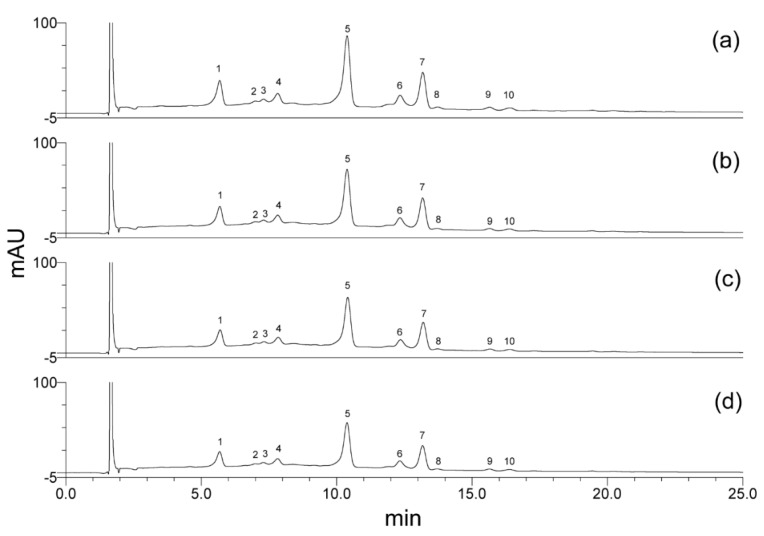
HPLC chromatograms of encapsulated blue corn powders anthocyanins obtained by spray drying at different agave fructan addition. (**a**) 6%, (**b**) 8%, (**c**) 10%, (**d**) 12%. Peak 1: cyanidin-3-glucosise, peak 3: pelargonidin-3-glucoside, peaks 2, 4, 5, 6, 7, 8, 9, 10: not identified.

The individual anthocyanin content is shown in Table 5. Cyanidin-3-glucoside and pelargonidin-3-glucoside contents significantly decreased (*p* < 0.05) as agave fructan concentration increased in encapsulated powders. This effect is clearly observed in their respective chromatograms (Figure 5), where the peaks 1 (cy-3-glu) and 3 (pg-3-glu), and also the unidentified peaks (2, 4, 5, 6, and 7), decreased when increasing the concentration of fructans. This decrease was similar to that found for total anthocyanins and it is related to a higher solids content, humidity, and a_w_, generated by fructans which could hydrate the anthocyanin structure (cation flavylium) and favor its degradation to colorless chalcone [41]. Also, the susceptibility of the identified anthocyanins (cy-3-glu and pg-3-glu) to the temperature caused by the spray-drying is clearly observed (Table 5) by comparison with the initial extract, with considerable losses (76.75%–90.16% for cy-3-glu and 80.04%–88.14 for pg-3-glu). Although the spray-drying decreased the individual anthocyanins considerably, the 10 peaks detected in the extract (Figure 4), were detected in all the powders (Figure 5), even in the sample with 12% of carrier. According to the results of individual anthocyanins and in terms of pigment and antioxidant content, the powders with the best results were those obtained with 6% of fructans.

## 4. Conclusions

This study describes the use of agave fructans in different concentrations as a wall or carrier material for the encapsulation of blue corn extract using the spray-drying technique. The results of encapsulated powders of blue corn extract indicated that the increase of agave fructans increased the bulk density, humidity, a_w_, pH, and WSI. Conversely, the hygroscopicity, WAI, as well as the total anthocyanin, total polyphenol, antioxidant activity, and individual anthocyanins contents decreased. Also, the color was affected with the increase in the concentration of fructans, increasing the luminosity and affecting this parameter (L*), mainly to the total color difference (ΔE) of the encapsulated powders. The glass transition temperature obtained (>50 °C) indicates a great stability of the products during storage. Morphological (SEM) properties were not affected by the different concentrations of fructans used. The results suggest that mixtures with agave fructans at concentrations of 6% (*w*/*v*) with extracts rich in anthocyanins from blue corn for encapsulation by spray-drying at 150 °C, favored the best values of anthocyanin (29.27%), polyphenols (73.03%), and antioxidant activity (42.57%) retention with adequate physical properties for maximum stability during storage. Encapsulated powders of blue corn extract using agave fructans as a carrier represent a promising food additive for their possible incorporation into foods and pigment them naturally, in addition to imparting antioxidant properties and presence of bioactive compounds (soluble fiber) that can benefit the health of consumers.

## Figures and Tables

**Table 1:** Characteristics of blue corn *(Zea mays* L.) extract.

Determination	Extract
°Brix	5.73 ± 0.11
Moisture (%)	95.97 ± 0.02
Water activity (a_w_)	0.99 ± 0.03
pH	1.79 ± 0.02
Total anthocyanins (mg/100 g d.b.)	412.97 ± 24.13
Total polyphenols (mg GAE/100 g d.b.)	2042.42 ± 51.28
Antioxidant activity (μmol TE/g d.b.)	93.57 ± 3.10
Color parameters	
L*	34.30 ± 0.03
a*	37.44 ± 0.07
b*	9.75 ± 0.04
Chroma*	38.69 ± 0.08
Hue°	0.254 ± 0.001

Means ± standard deviation. GAE, gallic acid equivalents; TE, Trolox equivalents; d.b., dry basis.

**Table 2:** Physical properties of blue corn anthocyanin encapsulated powders by spray-drying using agave fructans as a carrier agent.

Agave Fructans (% *w/v*)	Moisture (%)	Water Activity (a_w_)	Higroscopicity (%)	Bulk Density (g/cm^3^)	pH	WAI	WSI	T_g_ (°C)
6%	9.47 ± 0.39 ^b^	0.123 ± 0.005 ^c^	49.68 ± 0.92 ^a^	0.625 ± 0.008 ^b^	2.15 ± 0.005 ^c,d^	0.944 ± 0.02 ^a^	1.34 ± 0.02 ^d^	50.71 ± 0.48 ^a^
8%	10.03 ± 0.07 ^b^	0.141 ± 0.021 ^b,c^	46.34 ± 1.02 ^b^	0.640 ± 0.012 ^a^	2.16 ± 0.005 ^d^	0.765 ± 0.04 ^b^	1.38 ± 0.02 ^c^	53.55 ± 2.62 ^a^
10%	10.49 ± 0.27 ^a,b^	0.143 ± 0.016 ^b,c^	41.07 ± 1.07 ^c^	0.642 ± 0.004 ^a^	2.20 ± 0.032 ^b,c^	0.619 ± 0.01 ^c^	1.45 ± 0.02 ^b^	50.01 ± 1.08 ^a^
12%	11.07 ± 0.86 ^a^	0.156 ± 0.005 ^b^	38.17 ± 0.27 ^c,d^	0.647 ± 0.012 ^a^	2.23 ± 0.032 ^b^	0.554 ± 0.02 ^d^	1.48 ± 0.01 ^b^	52.15 ± 2.39 ^a^
Agave fructans	4.44 ± 0.39 ^c^	0.189 ± 0.010 ^a^	36.45 ± 0.53 ^d^	0.580 ± 0.007 ^c^	5.12 ± 0.017 ^a^	0.148 ± 0.01 ^e^	1.57 ± 0.01 ^a^	50.13 ± 0.51 ^a^

Means ± standard deviation of duplicate treatments. Values with different letter per column indicate significant difference (*p* < 0.05), using the Tukey test. WAI, water absorption index; WSI, water solubility index; Tg, glass transition temperature.

**Table 3:** Color parameters of blue corn anthocyanin encapsulated powders by spray-drying using agave fructans as a carrier agent.

Agave Fructans (% *w/v*)	L*	a*	b*	Chroma*	Hue°	ΔE
6%	36.79 ± 0.32 ^d^	36.25 ± 1.34 ^a^	13.62 ± 0.26 ^a^	38.73 ± 1.18 ^a^	0.359 ± 0.018 ^a^	5.04 ± 0.35 ^c^
8%	37.77 ± 0.94 ^c^	36.29 ± 0.64 ^a^	13.46 ± 0.25 ^a^	38.90 ± 0.48 ^a^	0.355 ± 0.006 ^a^	5.29 ± 0.64 ^c^
10%	40.09 ± 0.50 ^b^	36.26 ± 0.21 ^a^	13.79 ± 0.39 ^a^	38.80 ± 0.33 ^a^	0.363 ± 0.007 ^a^	7.11 ± 0.61 ^b^
12%	42.32 ± 0.46 ^a^	36.33 ± 0.30 ^a^	13.65 ± 0.24 ^a^	38.81 ± 0.21 ^a^	0.359 ± 0.008 ^a^	8.57 ± 0.47 ^a^

Means ± standard deviation of duplicate treatments. Values with different letter per column indicate significant difference (*p* < 0.05), using the Tukey test. ΔE, total difference of color.

**Table 4:** Correlation coefficients of blue corn anthocyanin encapsulated powders.

Determinations	Total Anthocyanins	Total Polyphenols
Total polyphenols	0.951 *	
Antioxidant activity	0.933 *	0.938 *

* Significant correlation (*p* < 0.05).

**Table 5:** Content of the main individual anthocyanins of blue corn encapsulated powders by spray drying using agave fructans as carrier agent.

Agave Fructans (% *w/v*)	Cy-3-glu (mg/100 g d.b.)	RP (%)	Pe-3-glu (mg/100 g d.b.)	RP (%)
6%	50.09 ± 4.60 ^b^	23.24	6.32 ± 0.46 ^b^	19.95
8%	38.70 ± 0.14 ^bc^	17.96	5.30 ± 0.04 ^b^	16.72
10%	30.69 ± 0.27 ^cd^	14.24	3.95 ± 0.04 ^c^	12.47
12%	21.19 ± 0.23 ^d^	9.83	3.75 ± 0.11 ^c^	11.85
Extract	215.50 ± 3.57 ^a^	100.0	31.70 ± 0.35 ^a^	100.0

Means ± standard deviation of duplicate treatments. Values with different letter per column indicate significant difference (*p* < 0.05), using the Tukey test. Cy-3-glu, cyanidin-3-glucoside; Pe-3-glu, pelargonidin-3-glucoside; d.b., dry basis; RP, retention percentage.
